# Academy of Medicine

**Published:** 1889-04

**Authors:** Wm. C. Krauss

**Affiliations:** Attica, N. Y.; 5 Rue Rollin, Paris


### *ACADEMY OF MEDICINE.*

(Paris, February 19, 1887.)

REPORTED BY DR. WM. C. KRAUSS, Attica, N. Y.

Prof. PÉAN reports, in collaboration with M. Gilbert Ballet and M. Gélineau, a case of *epilepsie partielle* (Jackson's epilepsy), caused by a cerebral tumor located in the motor zone, and the operation for removal by trepanation:

A young man of twenty-eight years had been subject to epileptiform attacks for the past six years, appearing at first every eight to ten days, later on becoming remittent, producing an *etat de mal.* For more than five years the patient was treated with the bromides with fair success. In December, 1888, the attacks became so frequent and so severe as to threaten life. They are characterized as follows: Beginning with a painful spasm of the right great toe, rigidity of the leg on the same side soon yielding to contractions of a tonic and clonic character, then propagating itself in the same manner to the arm and face of the right side. The loss of consciousness was not noted in every attack, but, when present, occurred at an advanced stage. During the intervals, a paresis of the right inferior extremity was noticed.

M. Ballet, likewise M. Gélineau, gave their opinion that the attacks were produced by a cerebral process situated in the motor zone of the right inferior member, or in its immediate vicinity. The differential diagnosis having established a cerebral tumor, in all probability, it was decided to trephine, open the dura mater in the vicinity of the motor zone of the right inferior member, determine the cause of compression, and, if circumstances permitted, to remove the tumor.

The symptoms indicated the presence of a tumor in the frontal and parietal ascendant convolutions on the left side, or in their immediate vicinity. There remained to determine the exact point for applying the trephine. M. Ballet determined this point in the following manner: A horizontal line was drawn from the left external orbital apophysis towards the occiput. At a point on this line, seven centimeters distant from the point of departure, a vertical line of three centimeters was drawn. The end of this line furnished the first point of location, corresponding to the inferior extremity of the sulcus of Rolando. To determine the direction of this sulcus, around which are located the motor centers, it was necessary to find its superior extremity. To determine this, a cord was placed vertically from one external auditory meatus to the other; on the median line of the cranium, a point forty-seven millimeters posterior to this cord was taken as the superior extremity of the sulcus of Rolando, and the two extremities were united. The position of the sulcus of Rolando thus determined, the upper part of this line, close to the median line of the cranium, was chosen as the point of operation. (The motor zone of the right inferior extremity corresponds to the upper part of the left frontal and parietal ascendant convolutions, and lobulus paracentralis.) The integument was incised at this point, care being taken to detach the periosteum at the same time. The bony plate was removed piece by piece, and the dura thus exposed to view appeared in a healthy condition. Upon incising the pia, a large tumor was found adhering to it. The tumor was of a yellowish colort closely adherent to the pia, and was diagnosticated as a fibro-lipoma. It was carefully removed piecemeal from the center towards the periphery. The wound was closed, following all antiseptic precautions, and was cicatrized by the tenth day.

From the day after the operation the attacks diminished in number, and presented only the equivalents of an attack. The operation was performed two and one-half months ago, and for the past two months the patient has had no manifestation of an attack.

This case is all the more interesting, as being the first one of its kind successfully performed in France.

5 Rue Rollin, Paris.

---

Single numbers of the Buffalo Medical and Surgical Journal can be obtained of Peter Paul & Brother, 420 Main street, where subscriptions will be received.

